# Strontium Regulates the Proliferation and Differentiation of Isolated Primary Bovine Chondrocytes *via* the TGFβ/SMAD Pathway

**DOI:** 10.3389/fphar.2022.925302

**Published:** 2022-05-27

**Authors:** Siqi Liu, Bingyu Shen, Juan J. Loor, Qianming Jiang, Yang Yuan, Yezi Kong, Panpan Tan, Fangyuan Zeng, Chenxu Zhao, Xiaoyan Zhu, Jianguo Wang

**Affiliations:** ^1^ College of Veterinary Medicine, Northwest A&F University, Xianyang, China; ^2^ Department of Animal Sciences, Division of Nutritional Sciences, University of Illinois, Urbana, IL, United States

**Keywords:** strontium, bovine chondrocyte, proliferation and differentiation, TGFβ, Smad3

## Abstract

The present study evaluated the effects of strontium (Sr) on proliferation and differentiation of chondrocytes isolated from dairy cows, and whether Sr exerts its effects *via* transforming growth factor *β* (TGFβ) signaling. The chondrocytes were isolated from patellar cartilage from newborn Holstein bull calves (*n* = 3, 1 day old, 38.0 ± 2.8 kg, fasting) within 15 min after euthanasia, and treated with different concentrations of Sr (0, 0.1, 1, and 10 μg/ml, as SrCl_2_·6H_2_O). After pretreatment with or without activin receptor-like kinase 5 (ALK5) inhibitor (10 μM SB-505124) for 4 h, chondrocytes were incubated with Sr for another 4 h. Overall effects of Sr were evaluated relative to NaCl as the control. In contrast, the 1 μg/ml Sr-treated group served as the control to determine effects of preincubating with SB-505124. Western blot and qRT-PCR were used for measuring expression of proliferation-, differentiation-, and TGFβ1-responsive factors. Data were analyzed using one-way ANOVA in GraphPad Prism 7.0. Incubation with all doses of Sr increased TGFβ1/ALK5-induced SMAD3 phosphorylation, and at 10 μg/ml it inhibited ALK1-induced SMAD1/5/9 phosphorylation. Expression of mRNA and protein of the proliferation-responsive factors type Ⅱ Collagen α1 (COL2A1) and aggrecan (ACAN) was induced by Sr at 1 μg/ml. In contrast, Sr at 10 μg/ml inhibited the expression of differentiation-responsive factors type Ⅹ Collagen α1 (COL10A1) and secreted phosphoprotein 1 (SPP1), and at 1 μg/ml it had the same effect on alkaline phosphatase (ALPL) mRNA and protein levels. Cells were stained with PI/RNase Staining buffer to assess cell cycle activity using flow-cytometry. Incubation with Sr at 1 and 10 μg/ml induced an increase in the number of cells in the S-phase, leading to an increase in the proliferation index. Incubation with SB-505124 inhibited phosphorylation of SMAD3. Abundance of ACAN and COL2A1 mRNA and protein was lower when cells were pre-incubated with SB-505124. Overall, data indicated that Sr promotes proliferation and inhibits differentiation of primary chondrocytes by directing TGFβ1 signaling towards SMAD3 phosphorylation rather than SMAD1/5/9 phosphorylation. Whether these effects occur *in vivo* remains to be determined and could impact future application of Sr as an experimental tool in livestock.

## Introduction

Strontium (Sr) belongs to the second major group of elements, along with Ca and Mg (Pilmane et al., 2017). Although present in trace amounts in the body, similar to Ca, Sr is also a bone-seeking element due to its similar physicochemical properties. Most of the Sr entering the body is absorbed by bones and teeth (Nielsen, 2004). The function of Sr in the context of bone relates to its role in promoting osteoblast-mediated bone formation and inhibiting osteoclast-mediated bone resorption (Querido et al., 2016).

In ruminants, it is well-established that Ca absorption from the gastrointestinal tract, Ca reabsorption from the kidney and mobilization of skeletal Ca stores help maintain Ca concentrations in the blood (Hernandez-Castellano et al., 2020). Thus, because Ca levels in the blood do not reflect intestinal absorption capacity, protocols for using Sr as a surrogate marker have been developed (Milsom et al., 1987; Khan et al., 2013). Studies with cows and sheep have reported a close correlation between the absorption rates of oral Sr and radioactive Ca, indicating that the Sr concentrations in the blood measured orally can serve as an index for Ca absorption capacity of the gastrointestinal tract of dairy cows and sheep (Hyde and Fraser, 2014; Hyde et al., 2019). Although these studies have provided information on the use of Sr, it is unknown to what extent (if any) Sr can affect other tissues in the body.

The transforming growth factor *β* (TGFβ) superfamily comprises more than forty members including TGFβ, activin, and bone morphogenetic protein (BMP) (Chen et al., 2012) all of which play pivotal roles in the metabolism, differentiation, proliferation, and survival of chondrocytes (van Caam et al., 2016; Woods et al., 2021). SMAD family member (SMAD)-dependent signaling is a classical pathway of the TGFβ family and it involves binding of TGFβ to its tetrameric receptor comprised of activin receptor-like kinase 5 (ALK5) and TGFβ type II kinase receptor dimers. As a result, the signal delivered into cells induces subsequent phosphorylation of the receptor-SMAD components, SMAD2 and SMAD3 (Zhu et al., 2016; Thielen et al., 2019). These receptor-SMAD complex binds to the common-SMAD, SMAD4, to trigger nuclear translocation of this whole complex to regulate gene transcription. Together with the phosphorylation of SMAD1/5/9 *via* ALK1, these events (at least in non-ruminants) regulate cartilage and bone development as well as homeostasis (Wu et al., 2016).

A previous study reported that Sr can regulate proliferation and differentiation of chondrocytes in rats by promoting the expression of TGFβ1 and TGFβ2 (Kong et al., 2018). However, it is unknown if Sr can mediate the TGFβ pathway to regulate chondrocyte proliferation and differentiation in the bovine. Thus, the main objective of the present study was to determine *in vitro* the effect of Sr on proliferation and differentiation of bovine chondrocytes *via* the TGFβ signaling pathway.

## Materials and Methods

### Ethics Statement

This study was conducted at one of the experimental farms of Northwest A&F University (Shaanxi Province, China) in Western China (106˚55′57″E, 34˚48′41″N). The protocol was approved by the Animal Welfare and Research Ethics Committee at Northwest A&F University (Permit Number: 2021049), Shaanxi, People’s Republic of China.

### Animals and Tissue Collection

Tissue was isolated from newborn Holstein bull calves (*n* = 3; 38.0 ± 2.8 kg BW). At each of 3 consecutive d, within 15 min after euthanasia by a veterinarian with barbiturate, patellar cartilage was separated from the articular knee of each calf through surgical patellar excision. Patellar cartilage was then washed three times with 0.1% PBS and within 1 h transported to the laboratory on ice.

### Isolation and Culture of Bovine Primary Chondrocytes

Primary chondrocytes were isolated from patellar cartilage from each calf and cultured individually. Methods for isolation of chondrocytes were described in a previous study (Wang et al., 2013). Briefly, the cartilage was sliced into thin slices and incubated with 0.25% collagenase type II (1761015, Sigma, United States) at 37°C in 5% CO_2_ for 18 h. Cells were collected by passing through a 100-mesh filter and then centrifuged at 400 × *g* for 10 min with at least three washes. Chondrocytes were then cultured in a 60 mm culture dish (704001, Nest, China) in DMEM/High-glucose (12800017, Gibco, United States) with 10% fetal bovine serum (FB15015, Clark, United States) at 5 × 10^5^ cells/mL in a humidified atmosphere of 5% CO_2_ in air at 37°C. The culture medium was replaced regularly every 2 days. When the growth density of the cells reached about 80–90% confluence, cells were passaged using a 0.25% trypsin digestion solution (T1186, Gentihold, China). Cells from second passage were then used for further experiments.

For experiments, cells were treated with a solution of Sr chloride (SrCl_2_·6H_2_O, V900279, Sigma, United States) dissolved in 0.9% NaCl at different concentrations. In ruminants, the maximum concentration of Sr used to measure level of Ca absorption from blood is 1 μg/ml (Hyde et al., 2019). Thus, 1 μg/ml was set as the medium-dose group. Doses of 0.1 μg/ml and 10 μg/ml were used as low- and high-dose groups. Cells were cultured for 4 h and 0.9% NaCl was used as a control. To inhibit ALK5 kinase activity, we had another set of cells cultured with the ALK5 inhibitor SB-505124 (HY-13521, MedChemExpress, United States) at a concentration of 10 μM (Vogt et al., 2011; van Caam et al., 2017) for 4 h before treated with 1 μg/ml Sr. 1 μg/ml Sr-treated group served as a control.

### Toluidine Blue Staining and Immunofluorescence Staining

Second passage cells on glass coverslips were washed twice using PBS, stained with toluidine blue O (G3660, Solarbio, Beijing, China) for 5 min. Equal amounts of distilled water were then added and allowed to stand for 15 min. After washing twice with PBS, cells were observed and photographed using a microscope (Carl Zeiss GmbH, Jena, Germany).

Second passage cells on glass coverslips were rinsed three times using PBS and fixed with 4% paraformaldehyde for 30 min. After washing three times with PBS, cells were treated with 0.1% Triton X-100 diluted in PBS for 15 min at 37°C, and blocked with 5% BSA in PBS for 20 min. COL2A1 antibody (COL2A1, AF6528, Beyotime Biotechnology, China) was incubated overnight at 4°C, the glass coverslips rinsed and then incubated with Goat Anti-Rabbit IgG H&L (ab150077, Alexa Fluor 488) for 4 h at 37°C. Cell nuclei were counterstained with DAPI (C1002, Beyotime Biotechnology, China). Lastly, glass coverslips were observed and photographed using a fluorescence microscope (Carl Zeiss GmbH, Jena, Germany).

### Total RNA Extraction, Primer Design, and qRT-PCR

Total RNA was extracted using Trizol reagent (15596026, Invitrogen, Carlsbad, United States) following manufacturer’s protocols. Concentration and purity of RNA were checked with a NanoDrop 2000C (Thermo Scientific, Waltham, MA, United States). Samples had an optical density ratio at 260/280 nm > 1.9 and <2.2. Reverse transcription of the total RNA was conducted using SuperScript™ RT reagent kit (RR047A, TaKaRa, Japan). All primers used were designed with Primer Premier 6 (Premier Biosoft, United States) based on GenBank data. Primer sequences are listed in Table 1. The housekeeping gene *GAPDH* was used as an internal control. Quantitative real-time polymerase chain reaction (qRT-PCR) was performed using the 2 × M5 HiPer SYBR Premix EsTaq (MF787-01, Mei5bio, China).

**TABLE 1:** Sequences of the primers used in this study.

Gene^a^	NCBI reference sequence	Product length (bp)	Forward primer	Reverse primer
*ALK5*	NM_001130916	323	TTCCGTGAGGCAGAGATT	GCA​ATA​CAG​CAA​GTT​CCA​TT
*TGFβ1*	NM_001166068	159	GAC​ACC​AAC​TAC​TGC​TTC​A	ATC​CAG​GCT​CCA​GAT​GTA​A
*SERPINE1*	NM_174137.2	381	GGCTCAGACCAACAAGTT	TTC​ACC​TCA​ATC​TTC​ACC​TT
*ID1*	NM_001097568.2	73	GCT​CCG​CTC​AGC​ACT​CTC​AA	GAT​CGT​CCG​CTG​GAA​CAC​A
*ALK1*	NM_001083479.1	109	ACA​ACA​CAG​TGC​TGC​TCA​GAC​A	TGC​TCG​TGG​TAG​TGC​GTG​AT
*COL2A1*	NM_001001135.3	299	GTGGAAGAGCGGAGACTA	GGT​AGG​TGA​TGT​TCT​GAG​AG
*ACAN*	NM_173981	155	CGGAAGTGAGTGGAGAGT	GGT​GGT​GCT​GAT​GAC​AAT​A
*ALPL*	NM_176858.2	163	AAC​ACA​AGC​ACT​CTC​ACT​AT	GCC​ATC​TCT​ACC​ATC​TCA​G
*COL10A1*	NM_174634.1	184	AGC​TGA​GAT​CAT​GCT​GCC​AC	CTC​TCC​TCT​CAG​TGA​TAC​ACC​TTT
*SPP1*	NM_174187	158	AGA​GGA​GGA​CTT​CAC​ATC​A	TCA​GAT​TGG​AAT​GCT​TGT​TC
*VEGFA*	NM_001316955	270	CCTTGCTGCTCTACCTTC	TGG​TGA​TGT​TGA​ACT​CCT​C
*GAPDH*	NM_001034034	117	CCT​GCC​AAG​TAT​GAT​GAG​AT	AGTGTCGCTGTTGAAGTC

a ALK5, activin receptor-like kinase 5; TGFβ1, transforming growth factor β; SERPINE1, Serpin family E member 1; ID1, inhibitor of DNA binding 1; ALK1, activin receptor-like kinase 1; COL2A1, type Ⅱ Collagen α1; ACAN, aggrecan; ALPL, ALKaline phosphatase; COL10A1, type Ⅹ Collagen α1; SPP1, secreted phosphoprotein 1; VEGFA, vascular endothelial growth factor.

### Protein Extraction and Western Blotting

Total protein was extracted from chondrocytes using the radioimmunoprecipitation assay Lysis Buffer (P0013B, Beyotime Biotechnology, China), and concentration measured with the BCA Protein Assay Kit (P0012, Beyotime Biotechnology, China). Then, 50 μg protein were separated on a 10% or 8% bisacrylamide gel and transferred to a PVDF membrane (IPVH00010, Millipore, United States). Membranes were blocked with 5% skimmed milk or BSA solution in TBS-T buffer for 2 h, and incubated overnight at 4°C with antibodies including type Ⅱ Collagen α1 (COL2A1, 1:1000, AF6528, Beyotime Biotechnology, China), aggrecan (ACAN, 1:500, NB110-6524, Novusbio biologicals, United States), type Ⅹ Collagen α1 (COL10A1, 1:500, bs0554R, Bioss biotechnology, China), Osteopontin (OPN, 1:1000, also called SPP1, secreted phosphoprotein 1, bs0019R, Bioss biotechnology, China), vascular endothelial growth factor (VEGFA, 1:1000, NB110-2381ss, Novus Bio biologicals, United States), alkaline phosphatase (ALPL, 1:1000, DF6225, Affinity, United States), SMAD3 (1:500, NB100-56479ss, Novusbio biologicals, United States), pSMAD3 (1:1000, 9520T, Cell Signaling Technology, United States), SMAD1/5/9 (1:1000, AF0614, Affinity, United States), pSMAD1/5/9 (1:1000, 13820T, Cell Signaling Technology, United States) and runt-related transcription factor 2 (RUNX2, 1:1000, AF5189, Affinity, United States). Blots were incubated for 2 h in a horseradish peroxidase (HPR)-conjugated secondary antibody at 25°C. Membranes were detected using a chemiluminescence (ECL) system (ProteinSimple, Santa Clara, CA, United States). Results were analyzed using the ImageJ software (Media Cybernetics, Bethesda, MD, United States).

### Flow Cytometry

Cells were collected and stained with PI/RNase Staining Buffer (BD pharmingen) to measure cell cycle activity determined by flow cytometry (Coulter-XL). Data were analyzed in ModFit 3.0 (Verity Software House, ME, United States) and the proliferation index (PI) was calculated using the following equations (Ming et al., 2019):

PI=(S+G2/M)/(S+G2/M+G0/G1)

### Coomassie Blue Staining of the Cytoskeleton

Second passage cells in a 35 mm culture dish were cultured at a density of 5.0 × 10^4^ cells/mL. After treatment, cells were washed three times with 0.1% PBS and 1% TritonX-100 was added to each dish for incubation in a humidified 37°C incubator for 15 min. The dish was washed three times with buffer M (60 mM imidazole, 50 mM KCl, 0.5 mM MgCl_2_, 0.1 mM EDTA, 1 mM EGTA, 1 mM β-mercaptoethanol, pH7.5) to stabilize the cytoskeleton. Cells were then fixed in 3% glutaraldehyde for 30 min and washed three times with 0.1% PBS after fixation, followed by addition of 0.2% Coomassie blue R_250_ for 30 min. Cells were washed with running water and observed and photographed using a microscope (Carl Zeiss GmbH, Jena, Germany).

### Statistical Analysis

Each experiment was repeated at least 3 times on consecutive days. The data were analyzed using the GraphPad Prism 7.0 (GraphPad Software Inc., United States) software. All data are reported as means ± SEM. For data with dose-response effects to Sr, comparisons among groups were performed using one-way ANOVA with Dunnett’s multiple comparison test with 0.9% NaCl as the control group. For data with effects of preincubation with SB-505124, comparisons among groups were performed using one-way ANOVA with Dunnett’s multiple comparison test with 1 μg/ml Sr as the control. *p* < 0.05 was considered significant.

## Results

### Morphological Identification of Bovine Chondrocytes

**FIGURE 1:**
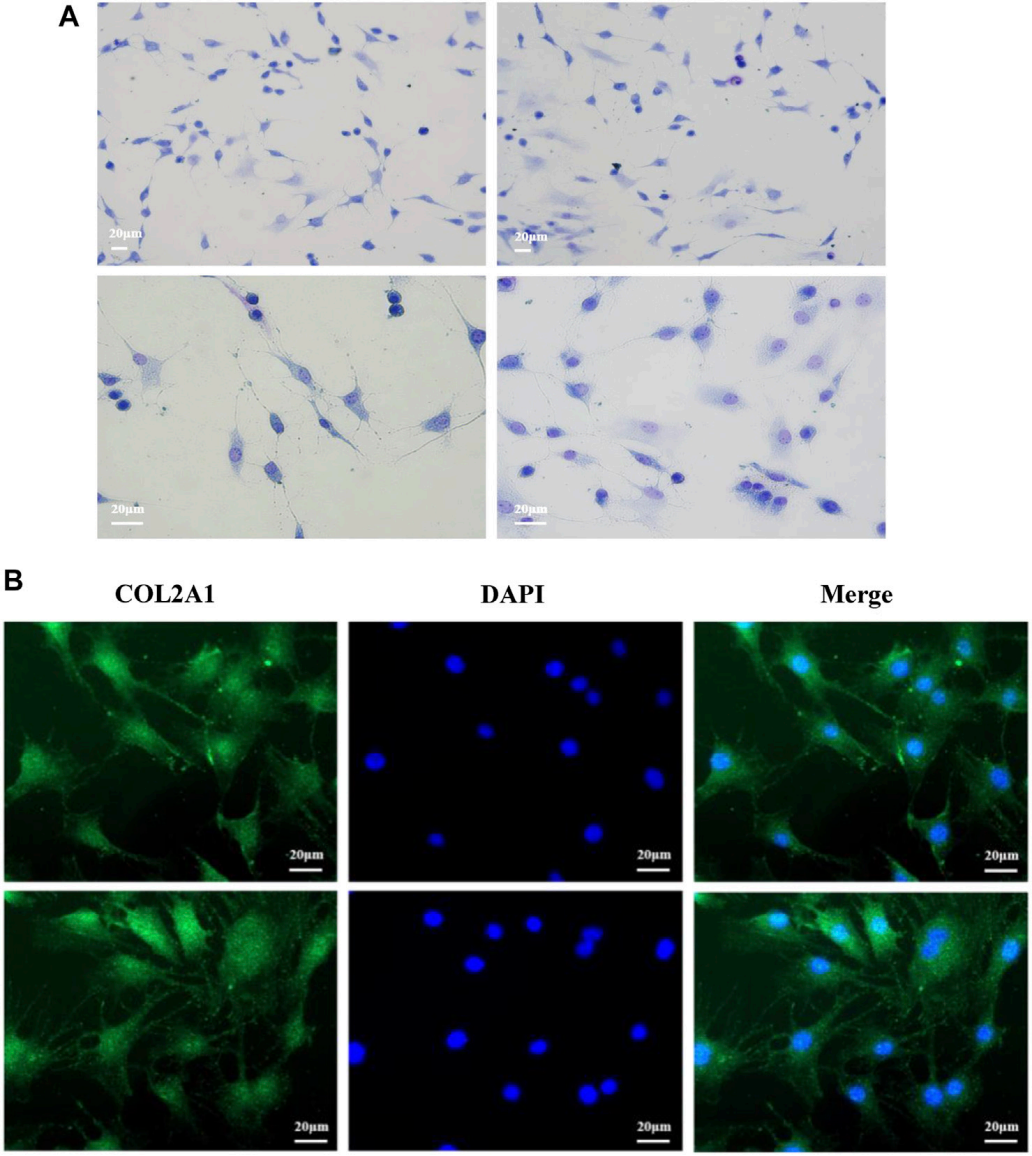
Morphological identification of bovine chondrocytes. **(A)** Toluidine blue staining of bovine chondrocytes. **(B)** Type II collagen α1 immunohistochemical staining.

Primary bovine chondrocytes were identified by Toluidine blue staining and type II collagen immunofluorescence staining. Chondrocytes were stained purple by toluidine blue (Figure 1A), and Type II collagen in chondrocytes stained green (type II collagen immunofluorescence). The nuclei stained blue when incubated with DAPI (Figure 1B). Together, these results indicated isolated cells from articular cartilage were chondrocytes.

### Strontium Promotes Proliferation and Inhibits Differentiation of Chondrocytes *via* TGFβ/SMAD3

**FIGURE 2:**
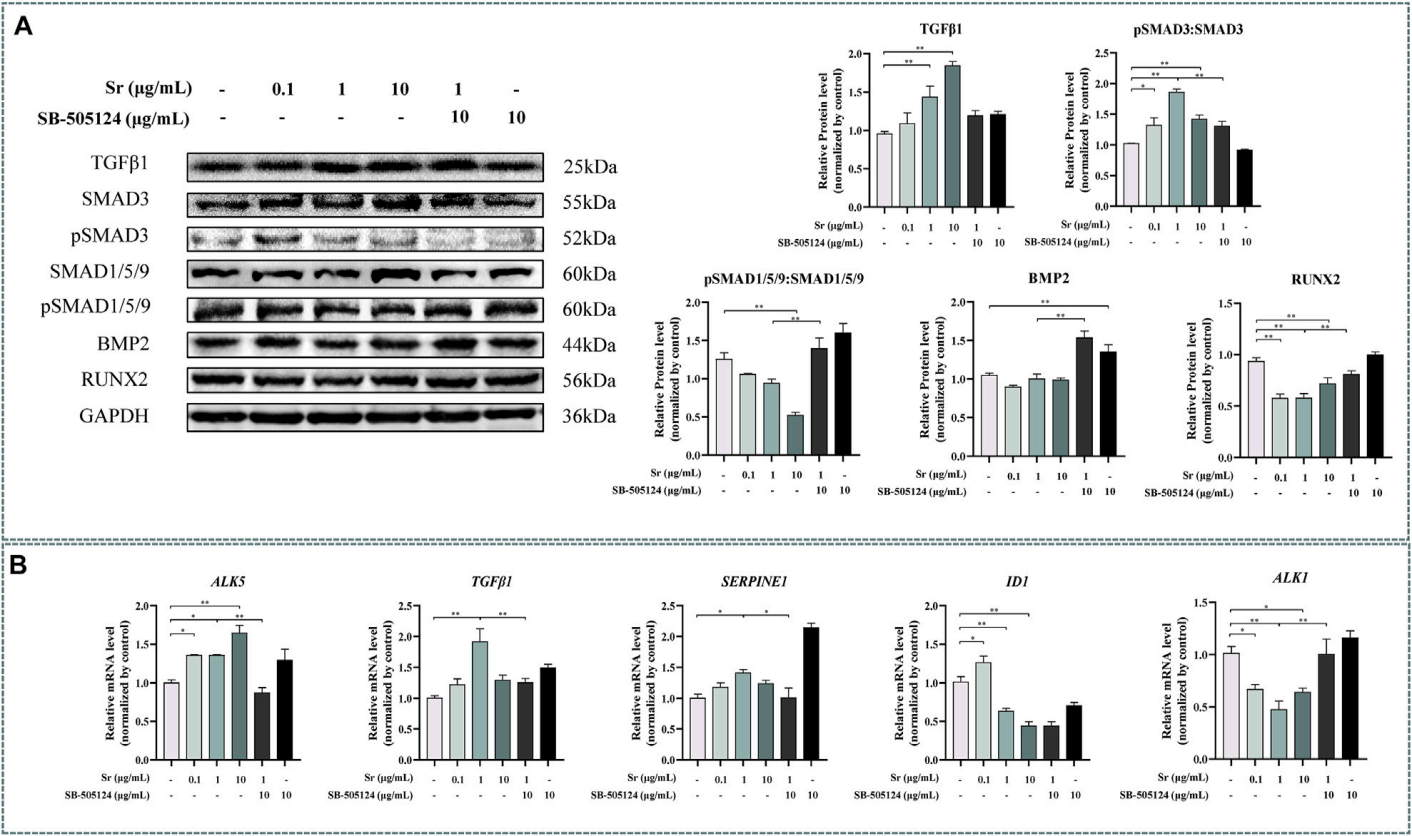
Effects of Sr and SB-505124 on the transforming growth factor *β* (TGFβ)/SMAD family member (SMAD) signaling pathway and downstream gene transcription in bovine chondrocytes. Chondrocytes were treated with different doses of Sr (0, 0.1, 1, 10 μg/ml) with or without activin receptor-like kinase 5 (ALK5) kinase inhibitor (10 μM SB-505124). **(A)** Western blot analysis and relative protein expression levels of SMAD3, pSMAD3, SMAD1/5/9, pSMAD1/5/9, TGFβ, bone morphogenetic protein 2 (BMP2), and runt-related transcription factor 2 (RUNX2). **(B)** mRNA expression levels of TGFβ1, serpin family E member 1 (SERPINE1), ALK5, ALK1, and inhibitor of DNA binding 1 (ID1). All experiments were repeated at least thrice. Data are means ± SEM (*n* = 3 in each group) *p < 0.05; **p < 0.01.

Exogenous Sr upregulated expression of TGFβ1 at both the transcriptional and protein level (Figure 2). Compared with the control, pSMAD3:SMAD3 ratio was higher (Figure 2A, *p* < 0.05) and the pSMAD1/5/9:SMAD1/5/9 ratio lower (*p* < 0.01) with each dose of Sr. Exogenous Sr did not alter BMP2 (*p* = 0.27 at 0.1 μg/ml; *p* = 0.97 at 1 μg/ml; *p* = 0.92 at 10 μg/ml, Figure 2A). Compared with the control, abundance of the key transcription factor RUNX2, regulated by pSMAD3 and pSMAD1/5/9, was decreased with each dose of Sr (Figure 2A, *p* < 0.01).

As evident from Figure 1B, abundance of the SMAD3-dependent genes *TGFβ1* and *ALK5* increased upon treatment with Sr at all doses (*p* < 0.05). Serpin family E member 1 (*SERPINE1*), another SMAD3-dependent gene, was greater with 1 μg/ml (*p* < 0.05). In contrast to the SMAD3-dependent genes, the abundance of inhibitor of DNA binding 1 (*ID1*) and *ALK1*, both SMAD1/5/9-dependent genes, decreased in all Sr-treated groups compared with the control (*p* < 0.05).

### Strontium Promotes Proliferation of Chondrocytes

**FIGURE 3:**
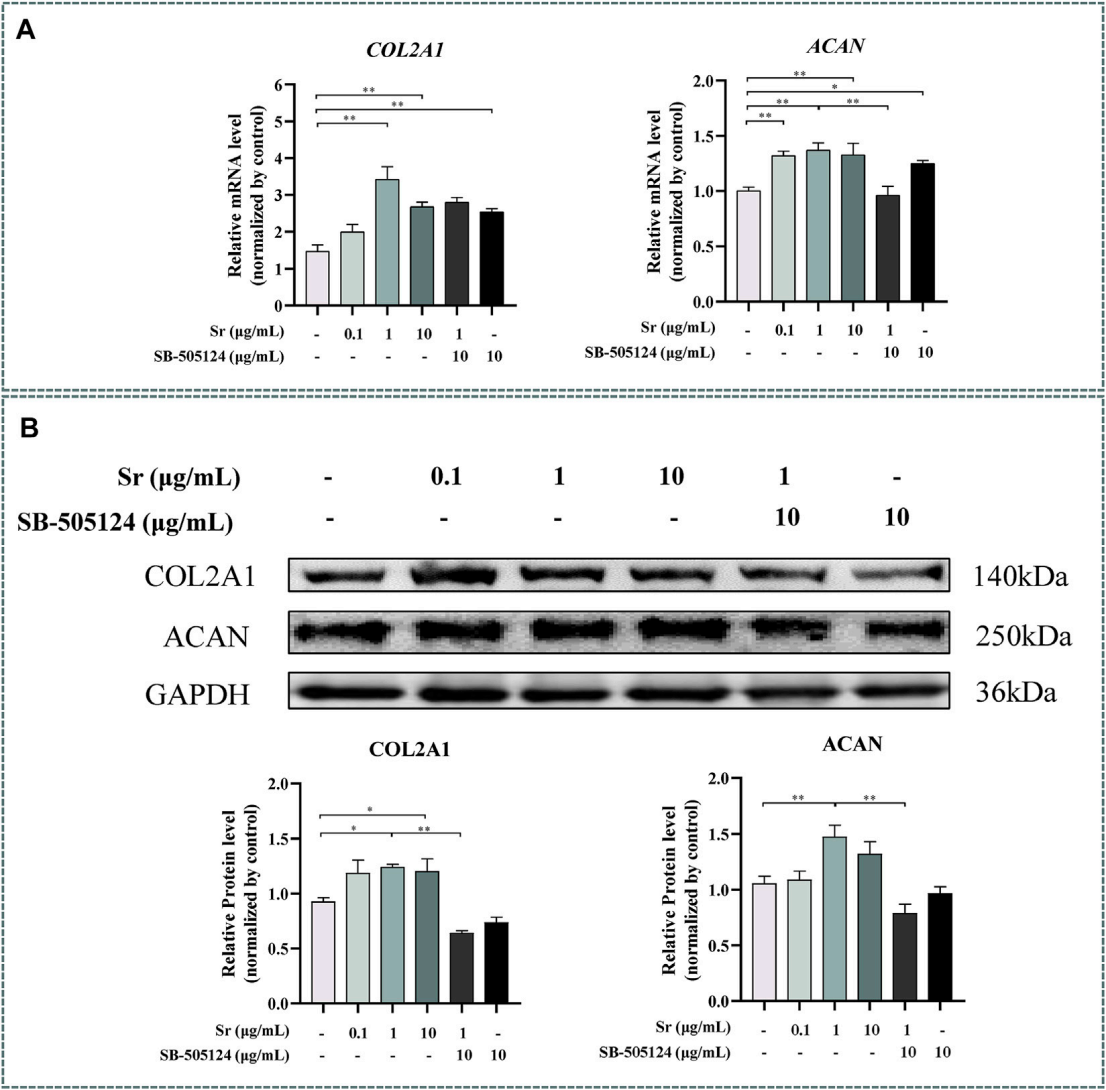
Effects of Sr and SB-505124 on proliferation-related factors in bovine chondrocytes. Chondrocytes were treated with different doses of Sr (0, 0.1, 1, 10 μg/ml) with or without activin receptor-like kinase 5 (ALK5) kinase inhibitor (10 μM SB-505124). **(A)** Relative mRNA expression levels of type Ⅱ Collagen α1 (COL2A1) and aggrecan (ACAN). **(B)** Western blot and protein expression levels of COL2A1 and ACAN. All experiments were repeated at least thrice. Data are means ± SEM (*n* = 3 in each group) *p < 0.05; **p < 0.01.

Both *COL2A1* and *ACAN* were upregulated by treating with Sr and reached the highest level in the 1 μg/ml group (*p* < 0.01) (Figure 3A). The upregulation of COL2A1 and ACAN was also confirmed at the protein level (Figure 3B), with upregulation of COL2A1 in the 1 and 10 μg/ml groups (*p* < 0.05) while ACAN was upregulated in the 1 μg/ml group (*p* < 0.01).

**FIGURE 4:**
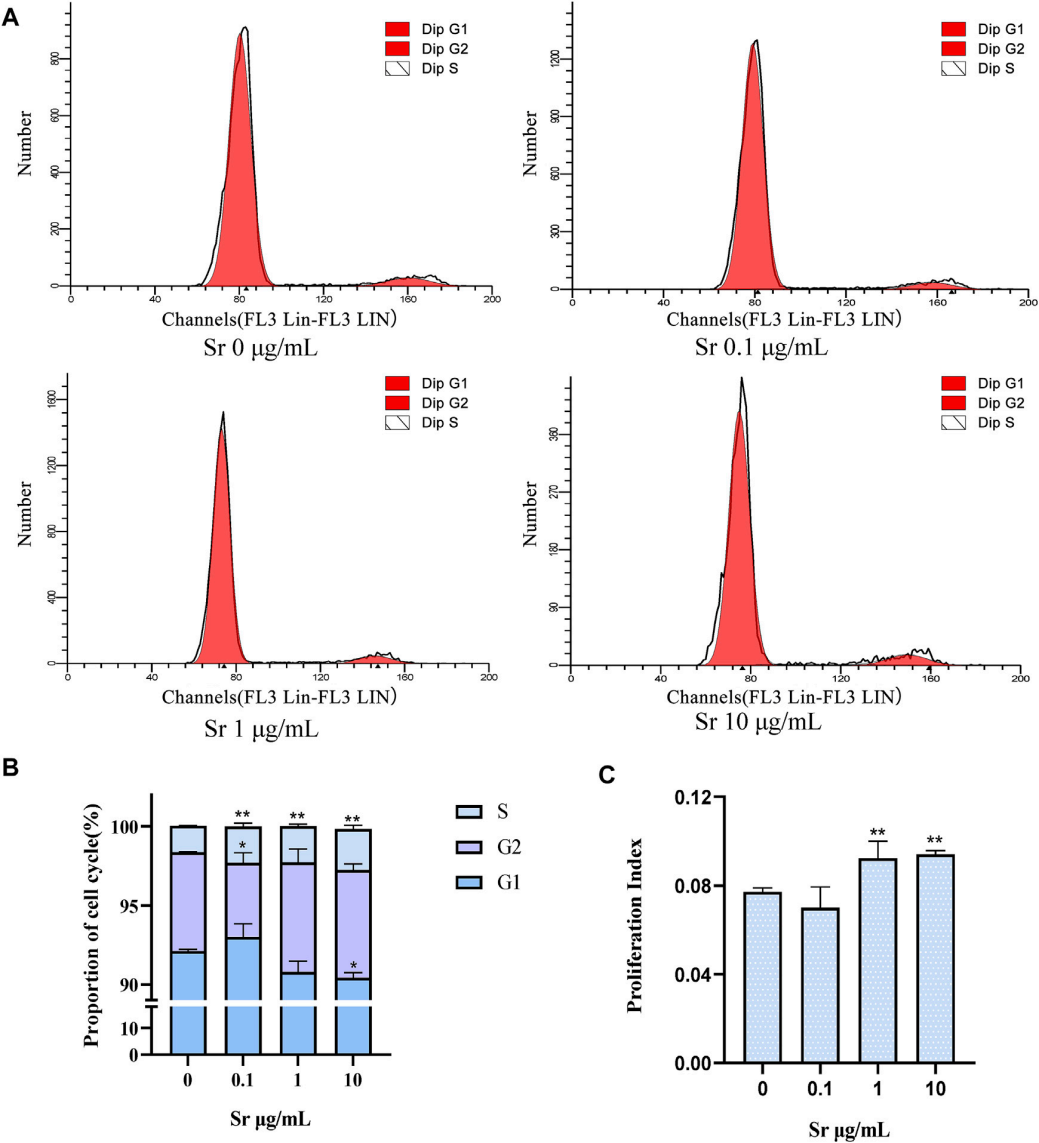
Cell cycle changes in bovine chondrocytes treated with different doses of Sr (0, 0.1, 1, 10 μg/mL). **(A)** Representative histograms of flow cytometry analysis of the cell cycle in bovine chondrocytes. **(B)** Representative images of cell cycle distribution. **(C)** Proliferation index (PI) was calculated based on the equation: PI = (S + G2/M)/(S + G2/M + G0/G1). Data are means ± SEM (*n* = 3 in each group) *p < 0.05; **p < 0.01.

**FIGURE 5:**
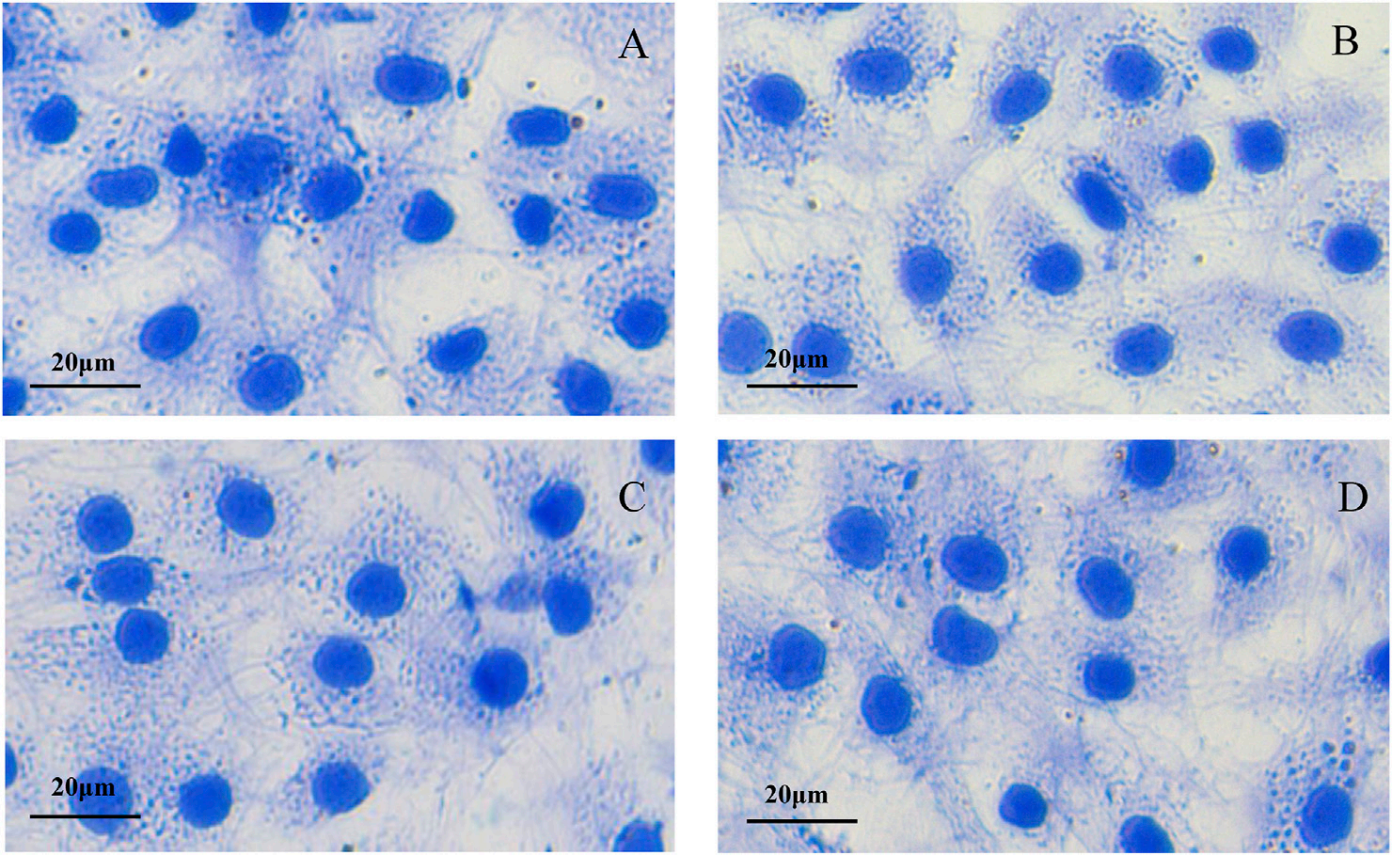
The effect of Sr on the cytoskeleton in bovine chondrocytes stained with Coomassie blue. **(A)** 0 μg/ml. **(B)** 0.1 μg/ml. **(C)** 1 μg/ml. **(D)** 10 μg/ml.

Compared with the control, treatment with Sr led to a remarkable increase in cells at the S-phase from 1.66 to 2.31% in the 0.1 μg/ml group to 2.27 and 2.59% in the 1 μg/ml and 10 μg/ml group (Figures 4A,B). Treatment with 10 μg Sr/mL decreased the number of cells in the G1 phase and 0.1 μg/ml Sr decreased cells in the G2 phase cells (Figures 4A,B). The proliferation index was higher in cells treated with 1 and 10 μg/ml Sr (Figure 4C). Culture with Sr had no effect on the cytoskeleton (Figure 5).

### Strontium Inhibits Differentiation of Chondrocytes

**FIGURE 6:**
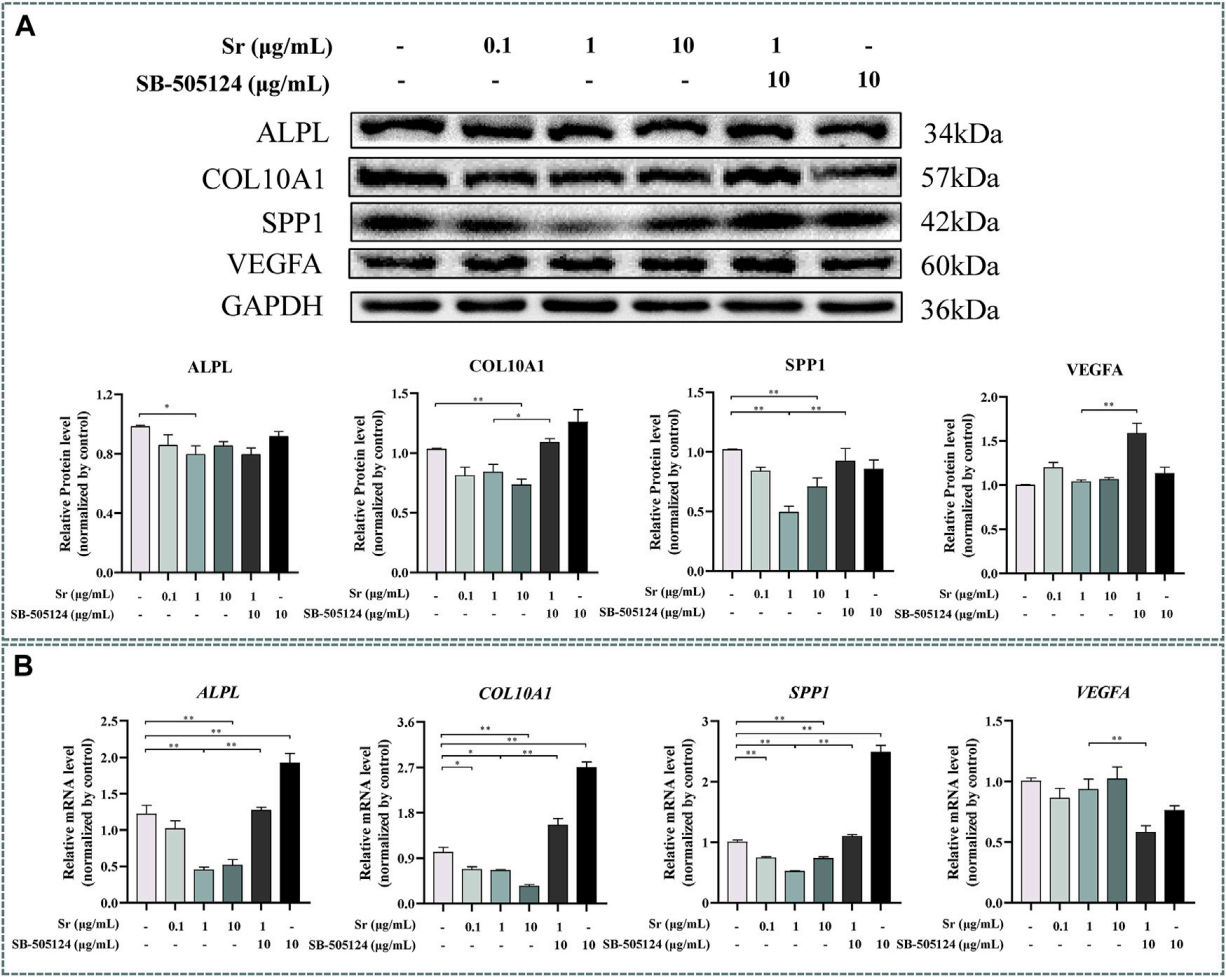
Effects of Sr and SB-505124 on differentiation-related factors in bovine chondrocytes. Chondrocytes were treated with different doses of Sr (0, 0.1, 1, 10 μg/ml) with or without activin receptor-like kinase 5 (ALK5) kinase inhibitor (10 μM SB-505124). **(A)** Western blot and protein expression levels of secreted phosphoprotein 1 (SPP1), alkaline phosphatase (ALPL), vascular endothelial growth factor (VEGFA), and type Ⅹ Collagen α1 (COL10A1). **(B)** mRNA expression levels of SPP1, ALPL, VEGFA, and COL10A1. All experiments were repeated at least thrice. Data are means ± SEM (*n* = 3 in each group) *p < 0.05; **p < 0.01.

Western blotting indicated that Sr treatment downregulated ALPL expression at a concentration of 1 and 10 μg/ml Sr (*p* < 0.05, Figure 6A). Expression of COL10A1 decreased in the 10 μg/ml treatment group, and expression of SPP1 decreased in 1 and 10 μg/ml Sr groups (*p* < 0.01, Figure 6A). Compared with the control, there was no effect of Sr on the expression of VEGFA. However, qPCR results revealed a marked downregulation of *ALPL* upon treatment with Sr at 1 μg/ml and 10 μg/ml (*p* < 0.01, Figure 6B). Both *COL10A1* and *SPP1* decreased in all Sr-treated groups (*p* < 0.05, Figure 6B). Dose of Sr had no effect on *VEGFA* expression.

### Strontium-Induced SMAD3 Phosphorylation

Pre-incubation with SB-505124 inhibited Sr-induced SMAD3 phosphorylation and promoted Sr-induced SMAD1/5/9 phosphorylation (*p* < 0.01, Figure 2A). SB-505124 inhibited mRNA expression of the SMAD3-dependent genes *TGFβ1*, *ALK5*, and *SERPINE1* (*p* < 0.05, Figure 2B). Although there was no significant change in *ID1* (*p* = 0.48), SB-505124 increased mRNA expression of *ALK1* (*p* < 0.01, Figure 2B).

Expression of *ACAN* mRNA decreased (*p* < 0.01, Figure 3A) while protein abundance of ACAN and COL2A1 decreased at the protein level when cells were pre-incubated with SB-505124 (*p* < 0.01, Figure 3B). Abundance of *ALPL*, *COL10A1*, and *SPP1* mRNA all increased (*p* < 0.01, Figure 6B) while protein expression of COL10A1 and SPP1 also increased (*p* < 0.05, Figure 6A).

## Discussion

Some studies have reported that Sr absorption could be used as a surrogate indicator for evaluating Ca absorption in the gastrointestinal tract in dairy cows and sheep (Hyde and Fraser, 2014; Hyde et al., 2019). However, the last few decades have witnessed studies on the effect of Sr in the cartilage and chondrocyte. For instance, Sr can reduce cartilage degeneration and promote ECM production in the ovariectomized rats (Mierzwa et al., 2017). Strontium gluconate was increased mRNA expression of *COL2A1* and *ACAN* in the osteoarthritic rat model (Hu et al., 2020). Thus, these studies provided some evidence for a role of Sr on the regulation of proliferation and differentiation of the cartilage. Unlike rodents, the role of Sr and the underlying molecular mechanisms on proliferation and differentiation of chondrocytes in ruminants are not well known. Further, whether Sr regulates chondrocyte proliferation and differentiation *via* the TGFβ pathway remains unclear. The present study demonstrated that Sr promoted TGFβ1/ALK5-induced SMAD3 phosphorylation and inhibited ALK1-induced SMAD1/5/9 phosphorylation. Sr promoted the expression of the proliferation-responsive factors COL2A1 and ACAN while inhibiting the expression of differentiation-responsive factors COL10A1, SPP1, and ALPL both at the mRNA and protein levels.

In non-ruminants, TGFβ1 is well-known to regulate proliferation and differentiation of chondrocytes (Wu et al., 2016). It plays an anti-hypertrophic role *via* the most classical ALK5/SMAD2/3-dependent pathway (Thielen et al., 2019), and several studies have reported that TGFβ1 also induces the SMAD1/5/9-dependent pathway *via* ALK1 (Chen et al., 2012; Charlier et al., 2019; Thielen et al., 2019). These two pathways have antagonistic functions in the chondrocytes (Finnson et al., 2008). For instance, a study in human chondrocytes reported that TGFβ1 could induce SMAD2 phosphorylation *via* ALK5 and SMAD1/5/9 phosphorylation *via* ALK1 (Finnson et al., 2010). It was also demonstrated that phosphorylation of SMAD2 increased the expression of COL2A1. Zhang et al. (2017) working with newborn mice reported that TGFβ regulated SMAD1/5/9 phosphorylation *via* the ALK1 in pulmonary artery smooth muscle cells and fibroblasts.

The significant increase of TGFβ1 levels in response to 1 μg/ml and 10 μg/ml Sr provided direct evidence that this mineral can affect TGFβ signaling. Furthermore, the present study determined that Sr, at a low concentration (i.e., 0.1 μg/ml), altered the pSMAD3:SMAD3 and the pSMAD1/5/9:SMAD1/5/9 ratio in a way that underscores its potential regulatory role of the TGFβ1 pathway. Such an effect is important in the context of biological responses that this mineral can induced in the bovine. For instance, Sr-containing α-calcium sulfate hemihydrate promoted osteogenic differentiation through TGFβ1-induced SMAD2/3 phosphorylation (Liu et al., 2019). In addition, in one of our previous studies we demonstrated that Sr can activate TGFβ1 signaling in rat chondrocytes (Kong et al., 2018). Overall, the results of the present study were consistent with the functional link between Sr and TGFβ signaling.

Existing studies have demonstrated that SMAD3 can be regulated by TGFβ through ALK5 (Li et al., 2005). This mechanism was confirmed in the present study when pre-treating chondrocytes with SB-505124 (Byfield et al., 2004; Vogt et al., 2011), the inhibitor of ALK5-mediated SMAD3 phosphorylation, inhibited SMAD3 phosphorylation in the co-treated group. Hellingman et al. (2011) reported that blocking SMAD2/3 with SB-505124 decreased abundance of COL2A1, which our results confirmed that COL2A1 and ACAN significantly decreased in the co-treatment group. In non-ruminants, BMP2, 4, 6, 7, and 9 are well-known to regulate SMAD1/5/9 *via* ALK1 (Li et al., 2004; Dexheimer et al., 2016). In addition, TGFβ also regulates SMAD1/5/9 *via* both ALK1 and ALK5 (Finnson et al., 2008; van Caam et al., 2017). Taking all these into account, although the present study did not detect effects of Sr on BMP2, any potential effects on BMP4, 6, 7, and 9 on the SMAD1/5/9 pathway cannot be excluded. Additional studies are needed to identify the mechanism whereby Sr can affect this pathway.

Previous studies in non-ruminants have reported that SMAD3-dependent genes such as *TGFβ1*, *SERPINE1*, and *ALK5* (van Caam et al., 2015) are upregulated in response to Sr. *ID1* and *ALK1* are well-known SMAD1/5/9-dependent genes in non-ruminants (Ehirchiou et al., 2010). Thus, the downregulation of *ID1* and *ALK1* at doses of 1 and 10 μg/ml Sr were in line with results demonstrating that Sr activated the SMAD3-dependent pathway while inhibiting the SMAD1/5/9-dependent pathway, the latter being a response already demonstrated in murine chondrocytes (Ehirchiou et al., 2010).

The differentiation and maturation of chondrocytes in non-ruminants is positively regulated, at least *in vitro*, by RUNX2 (Komori, 2018). This transcription factor promotes the expression of hypertrophy makers such as COL10A1, SPP1, VEGFA, and ALPL (Komori, 2017). Several studies reported a closed relationship between RUNX2 and SMAD proteins, and demonstrated that SMAD1/5/9 promotes chondrocyte maturation by stimulating the RUNX2 function, with SMAD3 serving to counteract RUNX2 (Wu et al., 2016; Yu et al., 2019). In the current study, the downregulation of RUNX2 in response to Sr was consistent with the downregulation of COL10A1 and SPP1. From these responses we speculate that SMAD3 and SMAD1/5/9 could at least partly regulate chondrocyte maturation *via* RUNX2. Support for this idea arises from data indicating that macrophages stimulated by 10 ng/ml IL-4 induced hypertrophy of human chondrocytes by promoting COL10A1 and RUNX2 (Ferrao Blanco et al., 2021). A role for RUNX2 on controlling hypertrophy of the tibial growth plate also was demonstrated in broilers (Wang et al., 2021). Overall, the present results were consistent with previous data.

**FIGURE 7:**
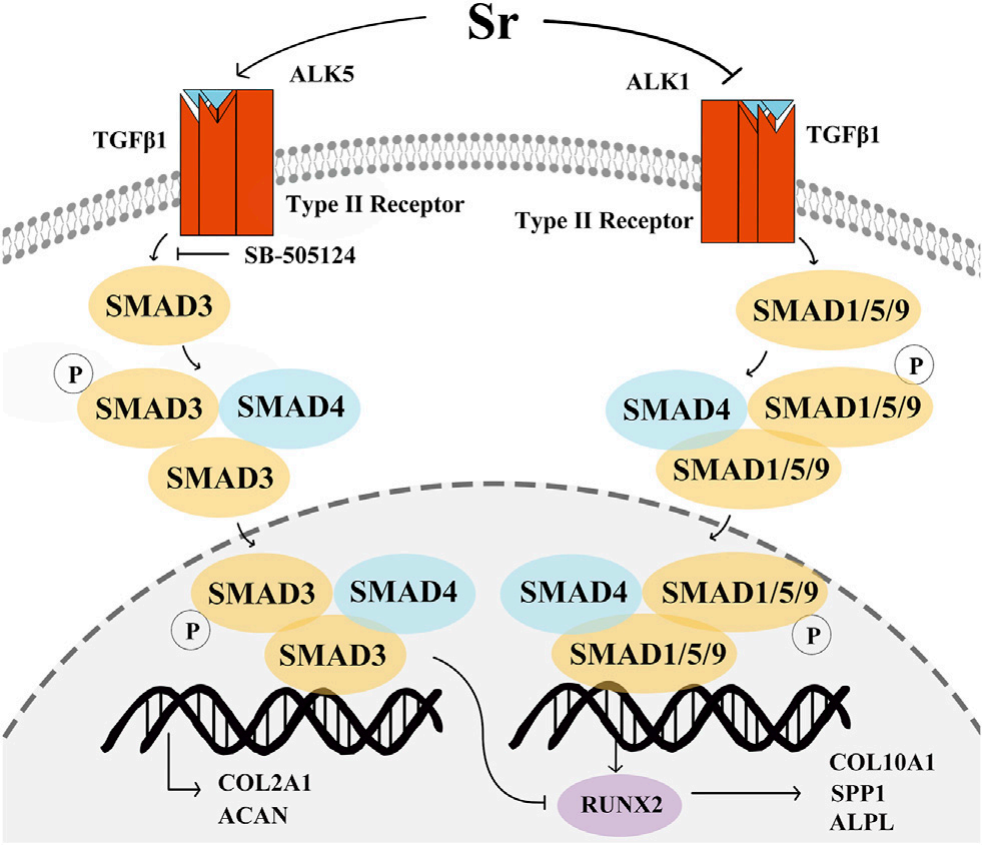
Schematic of the proposed mechanism whereby Sr affects proliferation and differentiation of bovine chondrocytes *via* the transforming growth factor *β* (TGFβ) signaling pathway. Sr shifts the signaling of TGFβ1 to SMAD family member 3 (SMAD3) by promoting activin receptor-like kinase 5 (ALK5) expression and inhibiting ALK1 expression. Phosphorylation of SMAD3 and SMAD1/5/9 after binding with SMAD4 transfers them into the nucleus to regulate gene transcription. An increase in pSMAD3 promotes the expression of type Ⅱ Collagen α1 (COL2A1) and aggrecan (ACAN). pSMAD1/5/9 induces type Ⅹ Collagen α1 (COL10A1), secreted phosphoprotein 1 (SPP1), vascular endothelial growth factor (VEGFA), and alkaline phosphatase (ALPL) by stimulating the function of runt-related transcription factor 2 (RUNX2). The increase in the pSMAD3 can inhibit the function of RUNX2 to decrease the COL10A1, SPP1, and ALPL expression.

The structural proteins COL2A1 and ACAN are upregulated in the proliferating zone during cartilage maturation, and when chondrocytes undergo hypertrophy, the proteins VEGFA, SPP1, COL10A1, and ALPL are secreted (Kronenberg, 2003; Kozhemyakina et al., 2015; Charlier et al., 2019; Vimalraj, 2020). In our previous study with rat primary chondrocytes there was a dose-dependent upregulation in COL2A1 with 1, 3 and 5 mM Sr (Wang et al., 2013). The fact that Sr promoted the expression of COL2A1 and ACAN at both mRNA and protein levels along with an increase in the PI suggested that Sr could promote chondrocyte proliferation in the bovine. Similarly, these results were also in agreement with data in human primary chondrocytes demonstrating that Sr chondroitin sulfate markedly upregulated the expression of COL2A1 and ACAN (Ma et al., 2017). The TGFβ co-receptor Cripto promoted COL10A1 by inducing SMAD1/5/9 signaling in ATDC5 cells and immortalized C28/12 human chondrocytes (Garcia de Vinuesa et al., 2021). A role for ALPL in the maturation and mineralization as well as the inhibitory effect of Sr on SPP1, COL10A1, and ALPL was reported in murine chondrocytes (Ehirchiou et al., 2020). Thus, together, available data suggest a mechanistic function for the systemic supply of Sr on fundamental aspects of chondrocyte development (Figure 7).

Similar to non-ruminants, Sr promotes SMAD3 phosphorylation and transcription of its downstream genes *via* the TGFβ1/ALK5 pathway, including COL2A1 and ACAN, both of which are key factors in the proliferation of chondrocytes. Differentiation of chondrocytes is controlled by Sr *via* decreasing the ALK1-induced SMAD1/5/9 phosphorylation and transcription of SPP1, COL10A1, and ALPL, all of which are key factors in differentiation. Overall, Sr activates TGFβ1-signaling towards phosphorylation of SMAD3 and, as such, systemic availability of this mineral can directly affect chondrocyte biology in dairy cattle.

## Data Availability

The original contributions presented in the study are included in the article/Supplementary Material, further inquiries can be directed to the corresponding author.
