# Supplementary figures and images for: Strontium Regulates the Proliferation and Differentiation of Isolated Primary Bovine Chondrocytes via the TGFβ/SMAD Pathway
Source: Front Pharmacol. 2022 May 27;13:925302. doi: 10.3389/fphar.2022.925302 (PMC9197245; doi:10.3389/fphar.2022.925302)

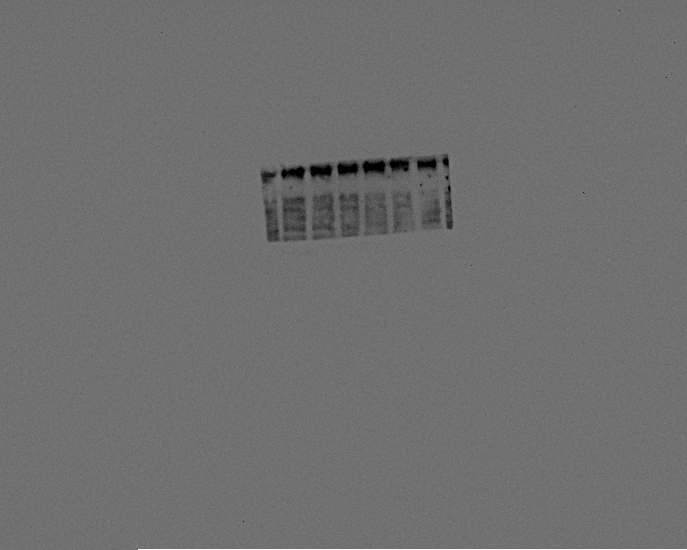

Supplement: Supplementary file 1 [file DataSheet1.ZIP › original western blot images/ACAN.tif]

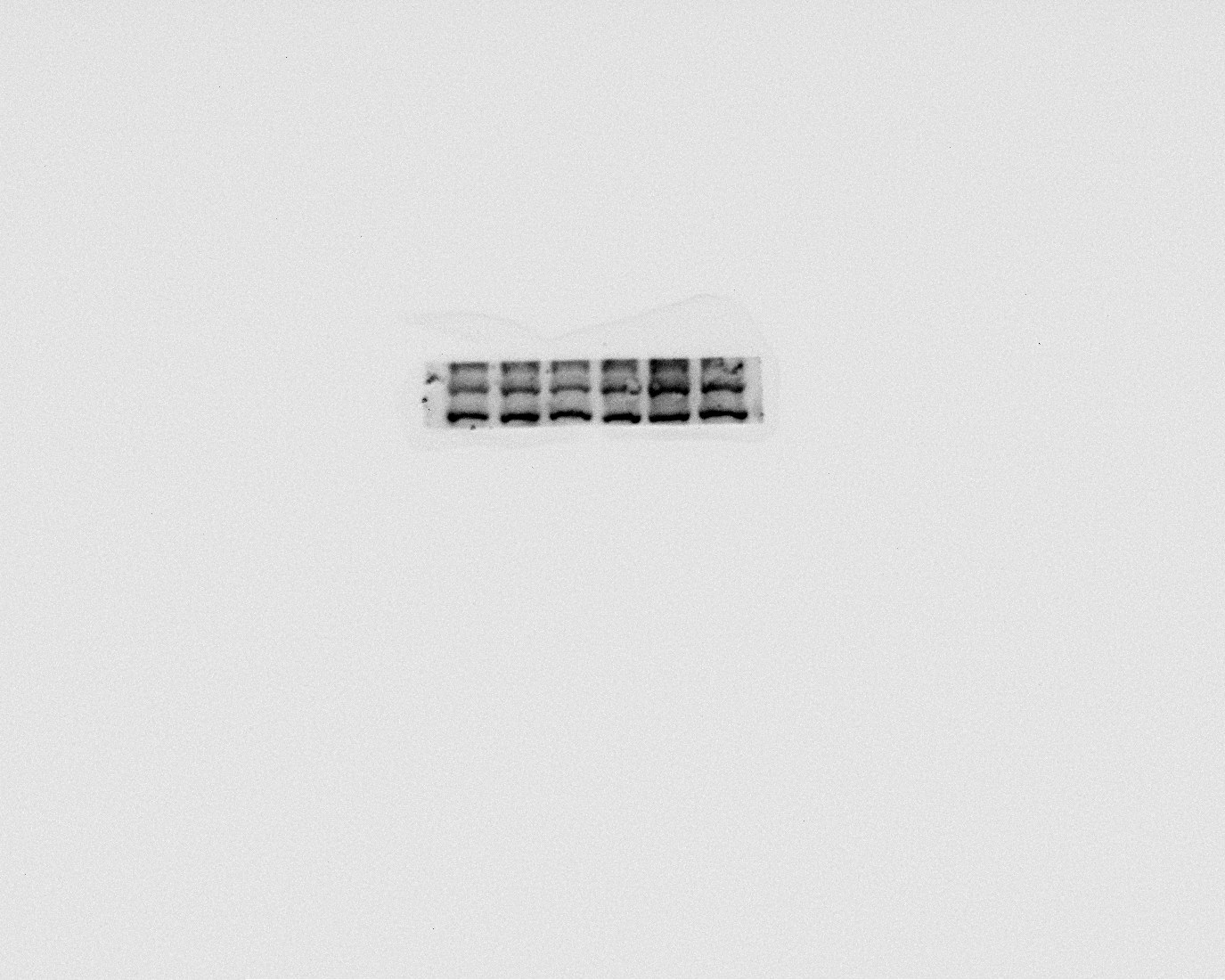

Supplement: Supplementary file 1 [file DataSheet1.ZIP › original western blot images/ALPL.tif]

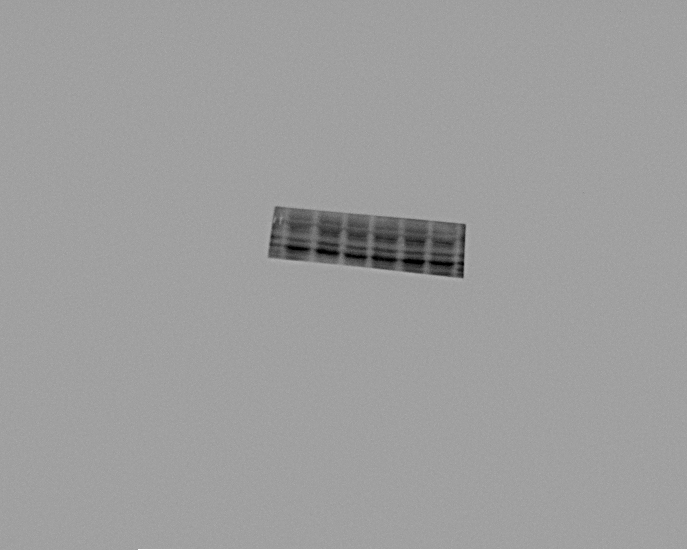

Supplement: Supplementary file 1 [file DataSheet1.ZIP › original western blot images/BMP2.tif]

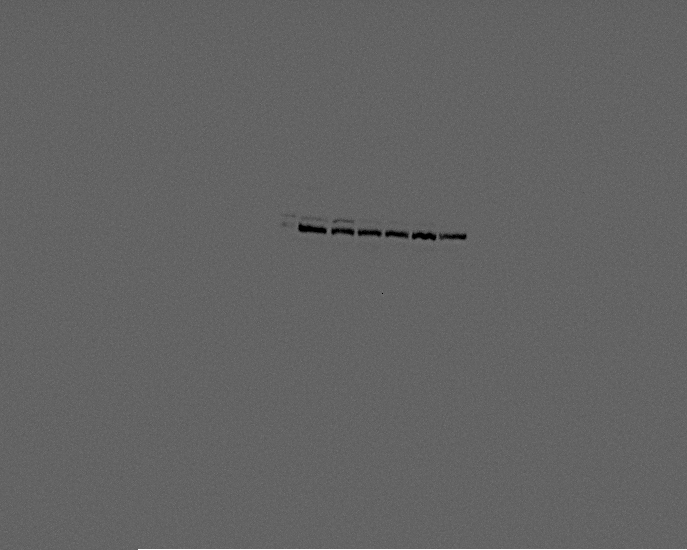

Supplement: Supplementary file 1 [file DataSheet1.ZIP › original western blot images/COL10A1.tif]

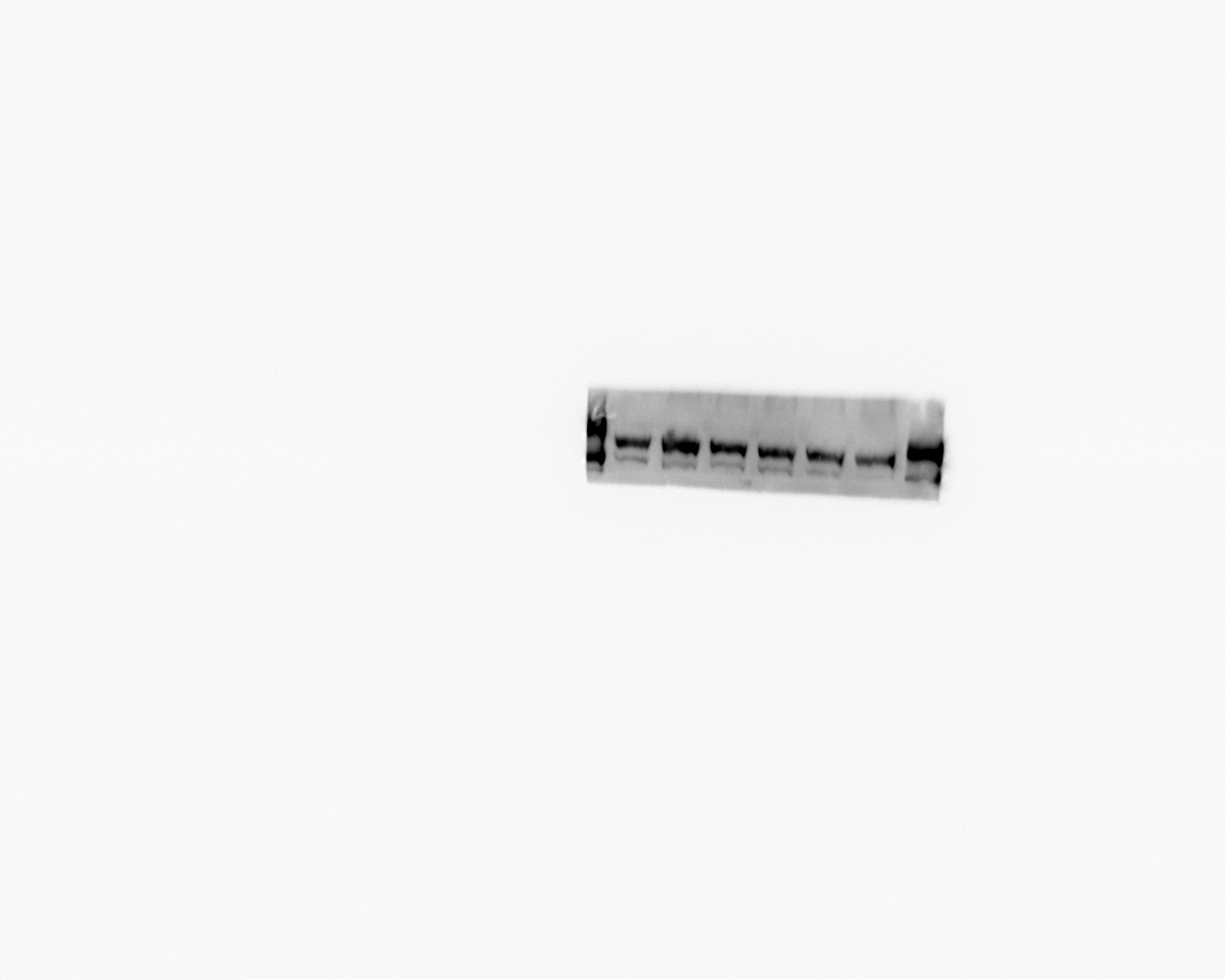

Supplement: Supplementary file 1 [file DataSheet1.ZIP › original western blot images/COL2A1.tif]

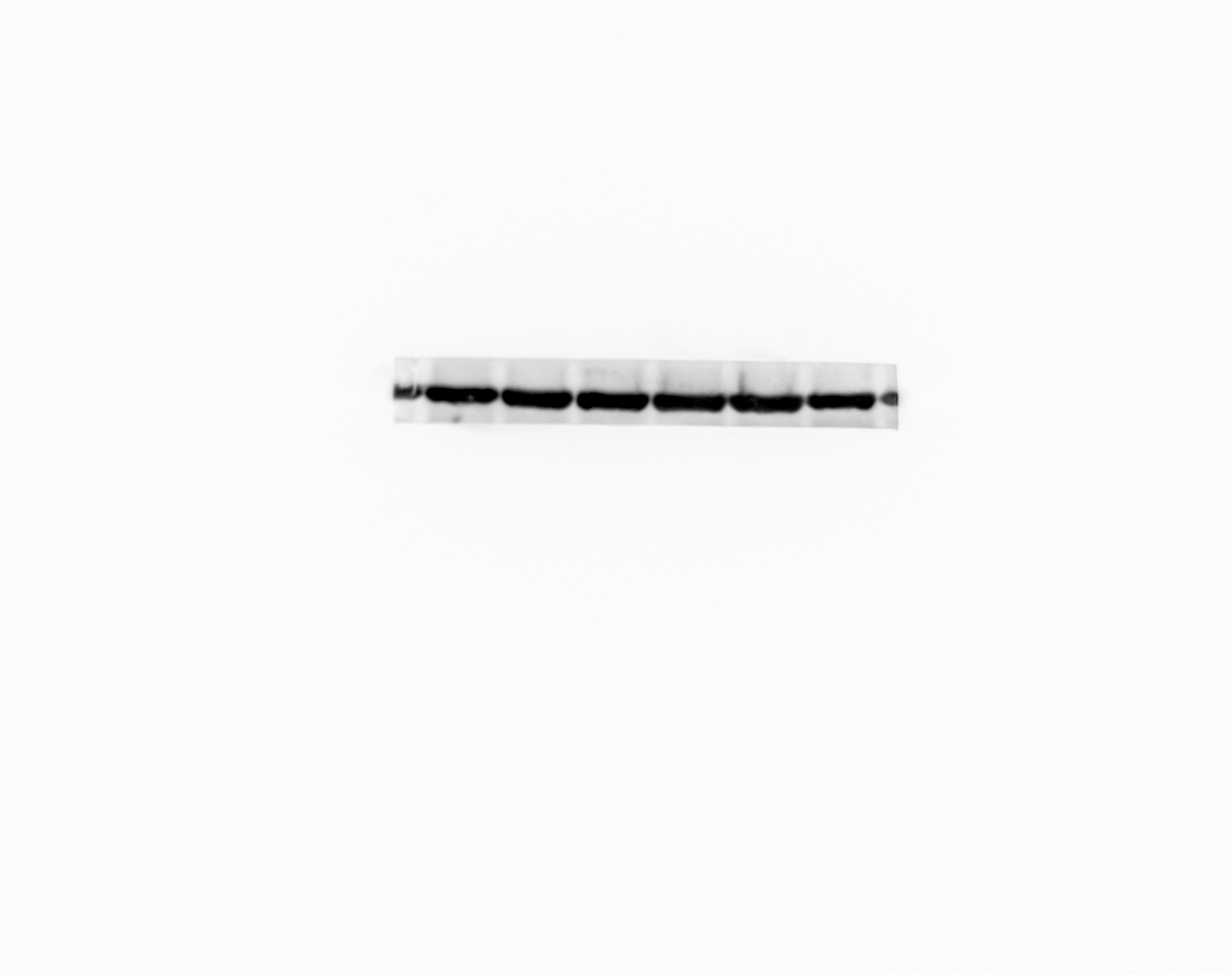

Supplement: Supplementary file 1 [file DataSheet1.ZIP › original western blot images/GAPDH.tif]

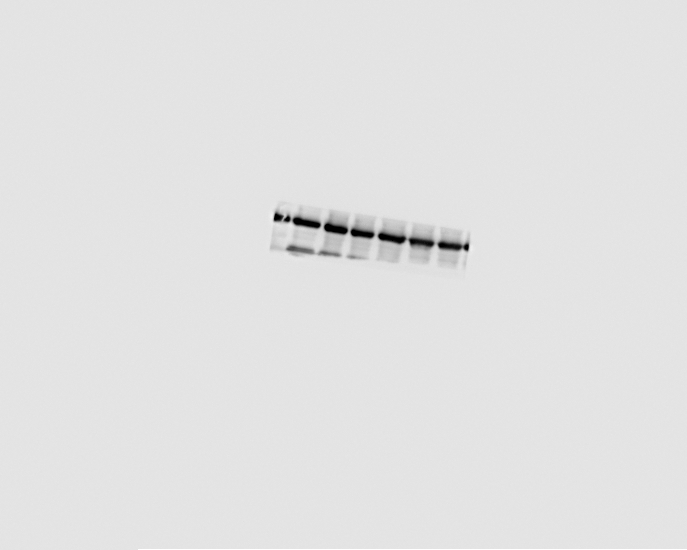

Supplement: Supplementary file 1 [file DataSheet1.ZIP › original western blot images/GAPDH2.tif]

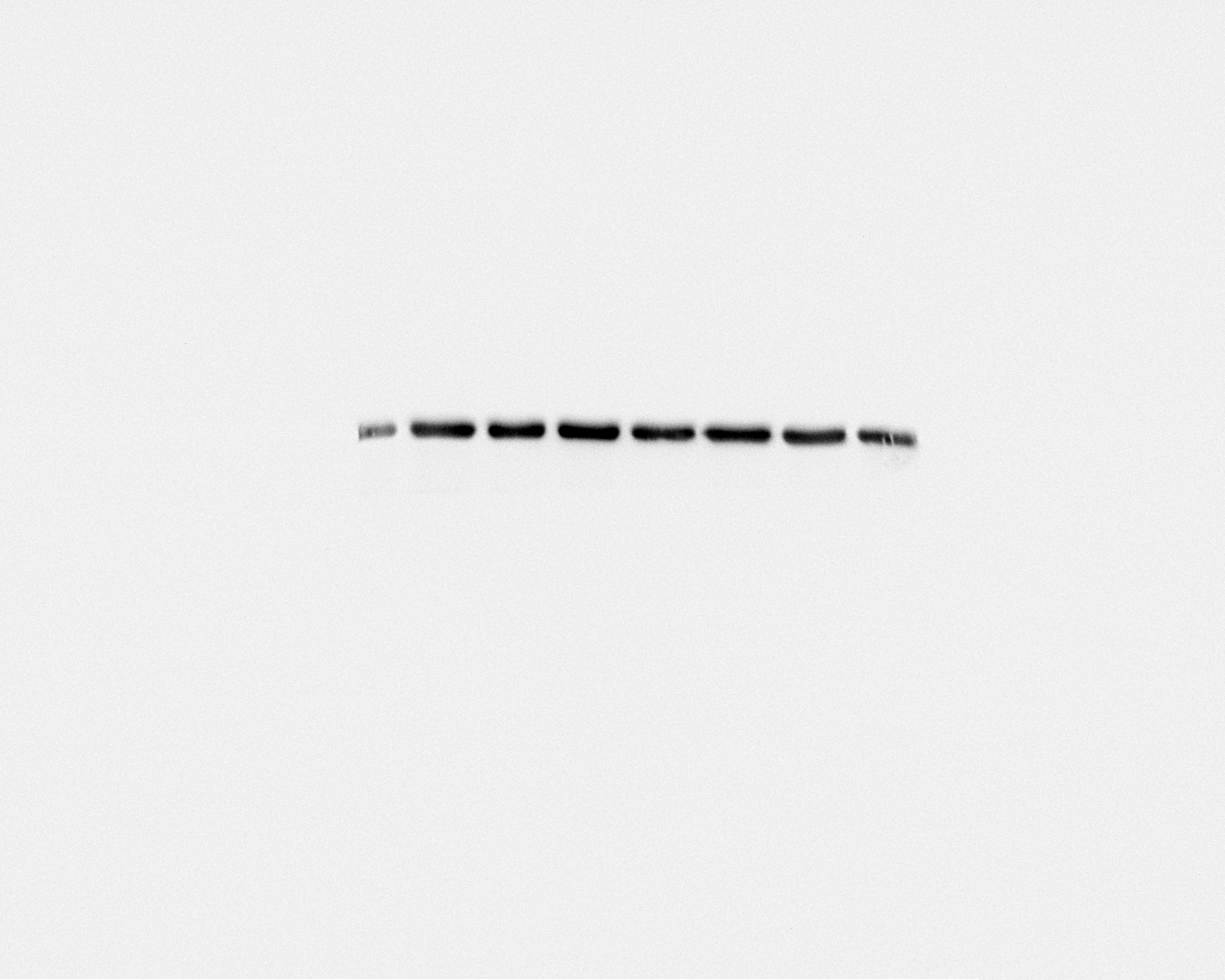

Supplement: Supplementary file 1 [file DataSheet1.ZIP › original western blot images/GAPDH3.tif]

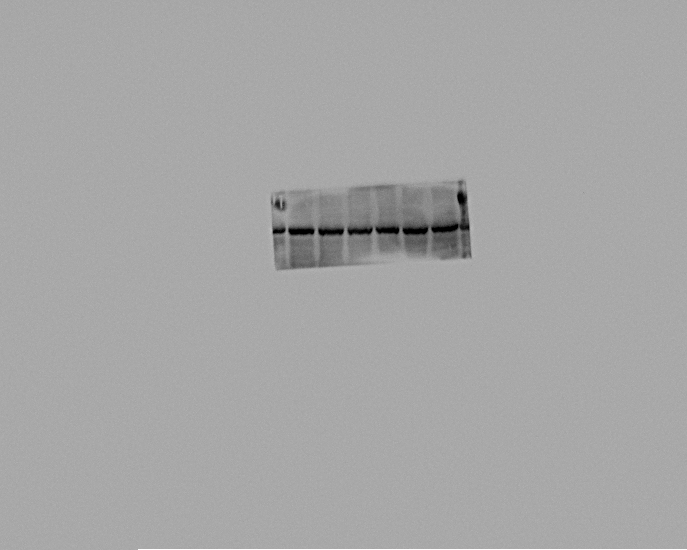

Supplement: Supplementary file 1 [file DataSheet1.ZIP › original western blot images/RUNX2.tif]

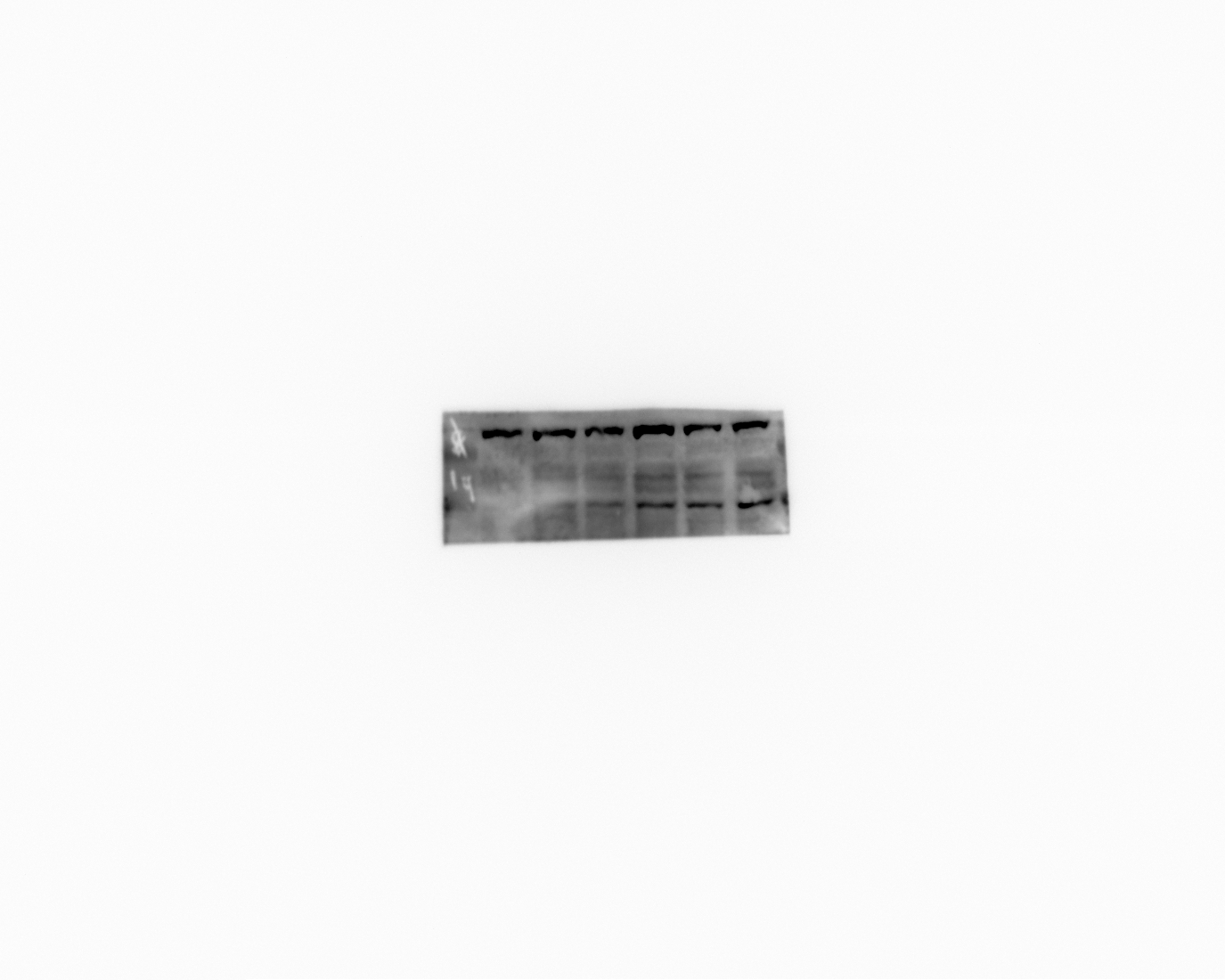

Supplement: Supplementary file 1 [file DataSheet1.ZIP › original western blot images/SMAD159.tif]

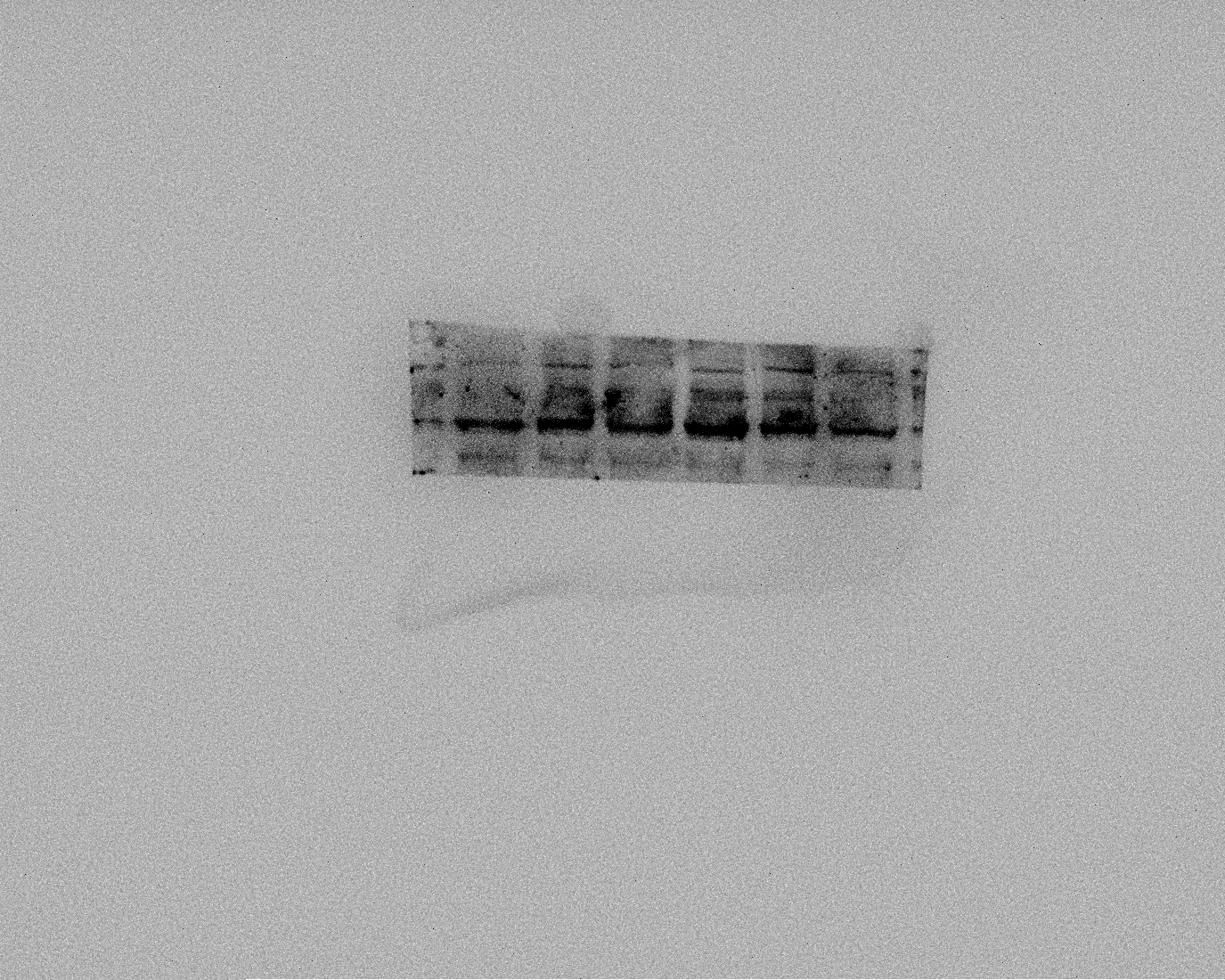

Supplement: Supplementary file 1 [file DataSheet1.ZIP › original western blot images/SMAD3.tif]

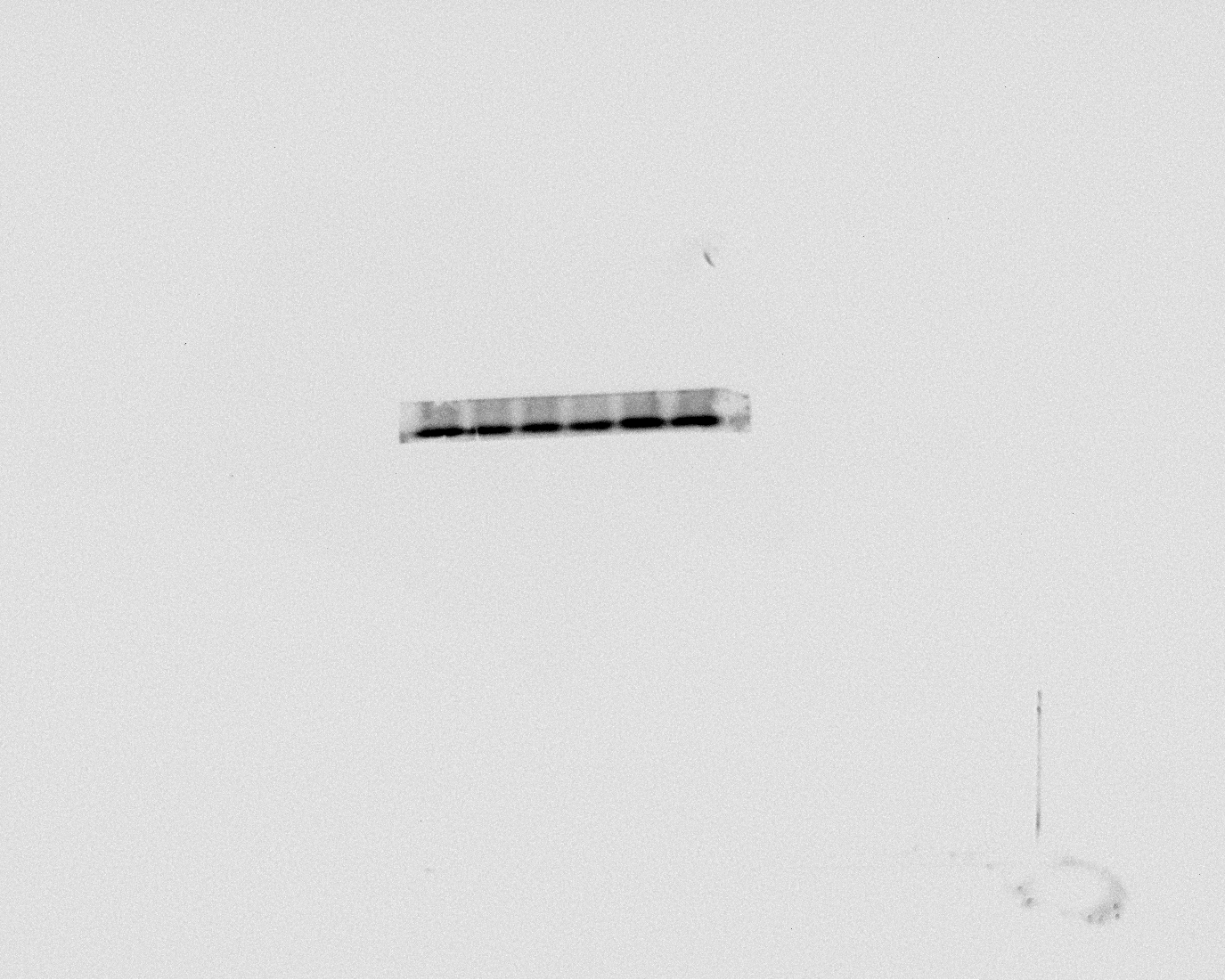

Supplement: Supplementary file 1 [file DataSheet1.ZIP › original western blot images/SPP1.tif]

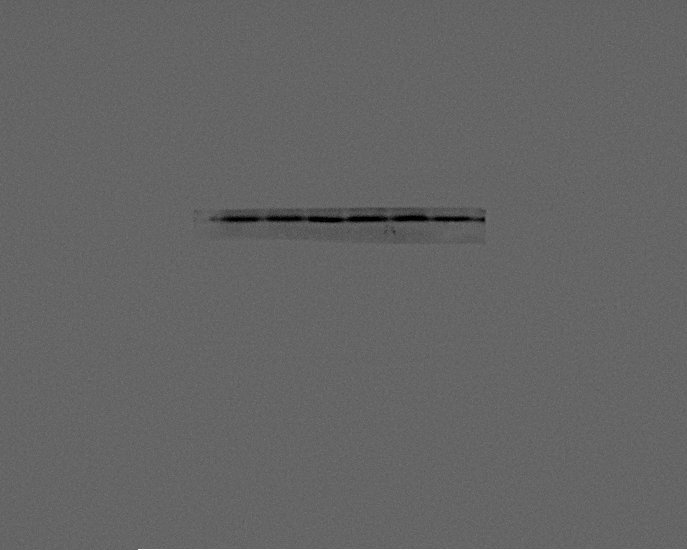

Supplement: Supplementary file 1 [file DataSheet1.ZIP › original western blot images/TGFb1.tif]

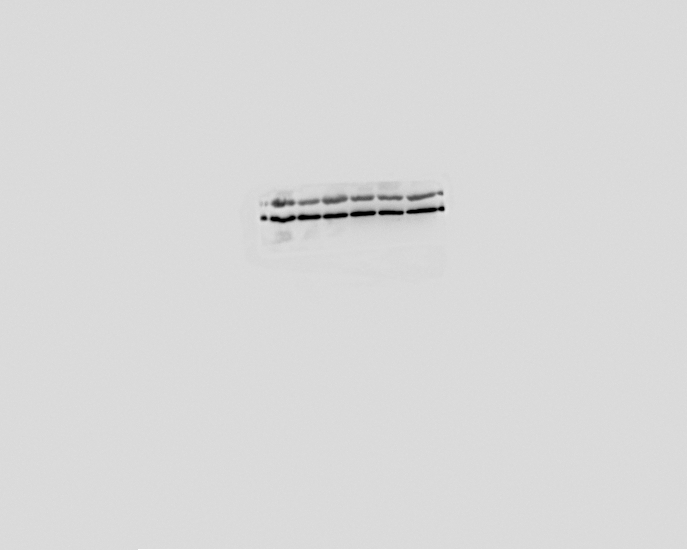

Supplement: Supplementary file 1 [file DataSheet1.ZIP › original western blot images/VEGFA.tif]

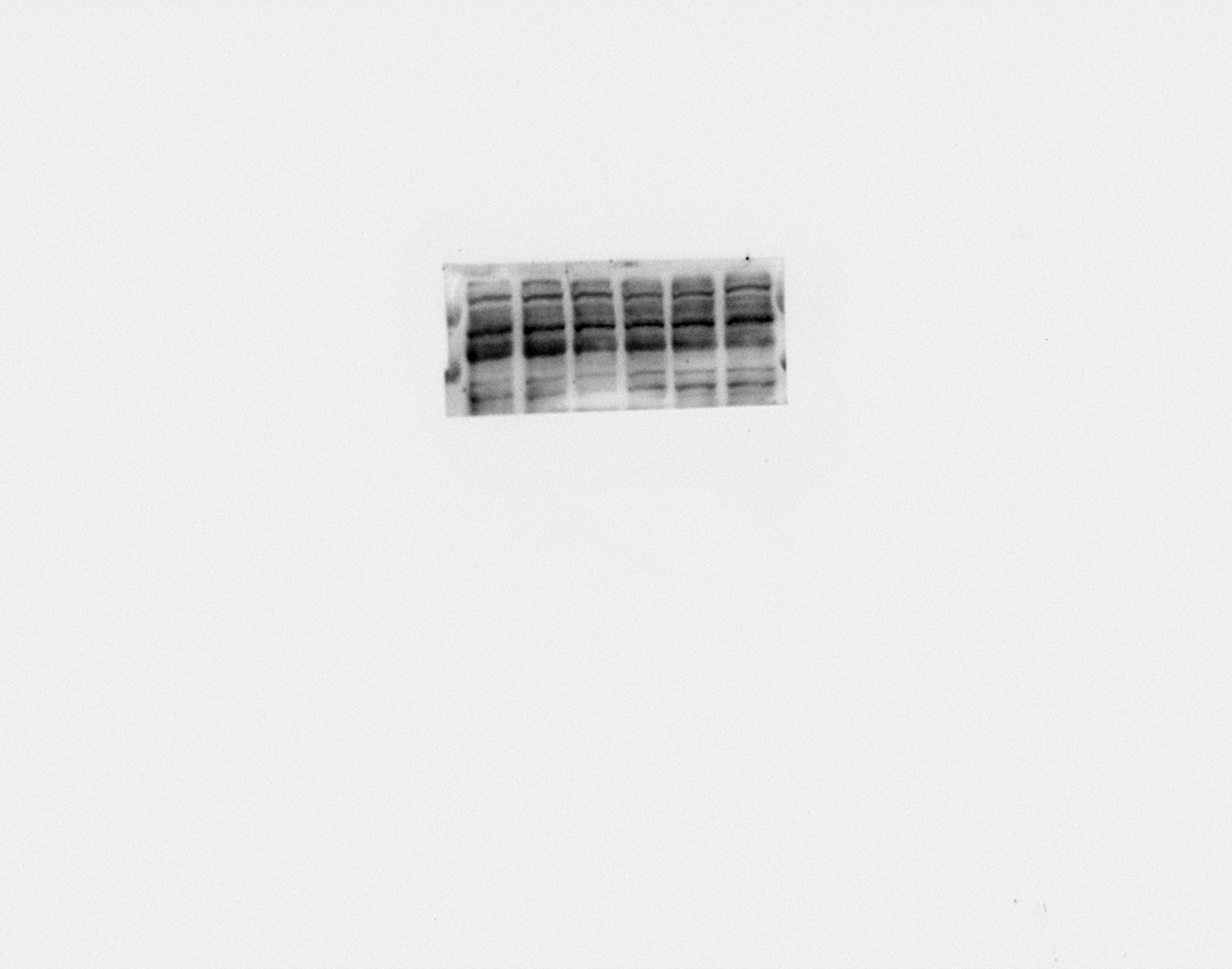

Supplement: Supplementary file 1 [file DataSheet1.ZIP › original western blot images/pSMAD159.tif]

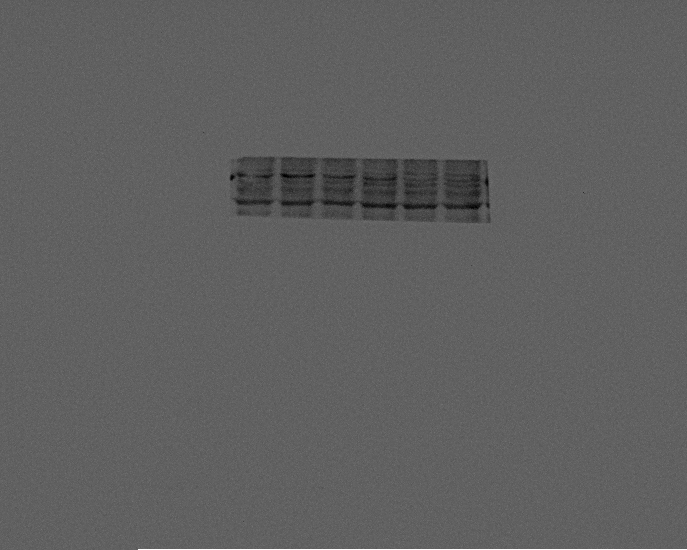

Supplement: Supplementary file 1 [file DataSheet1.ZIP › original western blot images/pSMAD3.tif]
